# Propolis and Their Active Constituents for Chronic Diseases

**DOI:** 10.3390/biomedicines11020259

**Published:** 2023-01-18

**Authors:** Vivek P. Chavda, Amit Z. Chaudhari, Divya Teli, Pankti Balar, Lalitkumar Vora

**Affiliations:** 1 Department of Pharmaceutics and Pharmaceutical Technology, L. M. College of Pharmacy, Ahmedabad 380008, India; 2 Department of Pharmaceutical Chemistry, L. M. College of Pharmacy, Ahmedabad 380009, India; 3 Pharmacy Section, L. M. College of Pharmacy, Navrangpura, Ahmedabad 380009, India; 4 School of Pharmacy, Queen’s University Belfast, 97 Lisburn Road, Belfast BT9 7BL, UK

**Keywords:** propolis, chronic kidney disease, rheumatoid arthritis, cancer, diabetes, metabolic syndrome and its associated chronic disease

## Abstract

Propolis is a mass of chemically diverse phytoconstituents with gummy textures that are naturally produced by honeybees upon collection of plant resins for utilization in various life processes in beehives. Since ancient times, propolis has been a unique traditional remedy globally utilized for several purposes, and it has secured value in pharmaceutical and nutraceutical areas in recent years. The chemical composition of propolis comprises diverse constituents and deviations in the precise composition of the honeybee species, plant source used for propolis production by bees, climate conditions and harvesting season. Over 300 molecular structures have been discovered from propolis, and important classes include phenolic acids, flavonoids, terpenoids, benzofurans, benzopyrene and chalcones. Propolis has also been reported to have diverse pharmacological activities, such as antidiabetic, anti-inflammatory, antioxidant, anticancer, immunomodulatory, antibacterial, antiviral, antifungal, and anticaries. As chronic diseases have risen as a global health threat, abundant research has been conducted to track propolis and its constituents as alternative therapies for chronic diseases. Several clinical trials have also revealed the potency of propolis and its constituents for preventing and curing some chronic diseases. This review explores the beneficial effect of propolis and its active constituents with credible mechanisms and computational studies on chronic diseases.

## 1. Introduction

Propolis emerged as a unique naturally derived resinous remedy possessing diverse medical importance that has gained popularity worldwide over the centuries [1]. Currently, tremendous clinical trials have begun to reveal the medicinal importance of propolis and push this remedy in pharmaceutical and nutraceutical areas. Honey bees contribute as pollinators in natural ecosystems and provide beeswax, propolis, royal jelly, venom and apitherapy as medicinal and nutritional resources for human health [2]. Propolis is loaded with diverse constituents, and this diversity varies depending on the type of honeybee species, plant source used for propolis production by bee, climate conditions and harvesting season [3,4]. The bioactive profile of propolis shows a correlation with ecosystems, especially the diverse variety of floral resources that play a key role in the survival and biodiversity of bees [5]. Over three hundred chemical components, including flavonoids, terpenoids, phenolic acids, benzofuranes, benzopyranes and chalcones, are major constituents of propolis which have biological activity [3]. Massive experimental trials have reported that propolis, especially that derived from Brazil, China, Taiwan and Iran, and its constituents have biomedical beneficiary activities, such as antidiabetic, anti-inflammatory, antioxidant, anticancer, immunomodulatory, antibacterial, antiviral, antifungal and anticaries activities [3,4,6]. Chronic diseases remain a global threat, with the most frequent being diabetes, chronic obstructive pulmonary disease (COPD), tuberculosis, cancer, cardiovascular disease (CVD) and chronic kidney disease (CKD), which also cause economic hardship worldwide [7]. Propolis and its pure bioactive compounds are reported to be effective and safe medicines as alternative therapies for chronic diseases [8]. In silico studies of Brazilian and Sulawesi propolis constituents with molecular docking promised propolis and its constituents as future drug candidates [9,10].

In this review, we first performed an exhaustive literature review from PubMed, Scopus, and SciFinder using the terms ‘propolis’, ‘propolis and chronic diseases’, ‘role of propolis constituents’, etc. From the search results and their analysis, we have identified the potential role of propolis constituents in chronic disorders. To support the same and evaluate the role of potential constituents, we carried out in silico studies using a molecular docking approach. We have attempted to summarize the research and clinical outcomes of such constituents of propolis for various chronic disorders and emphasize the therapeutic outcomes.

## 2. Propolis and Molecular Mechanism of Their Active Constituents

Propolis has over 300 molecular components, including polyphenols, steroids, vitamins and pollens, which justify its effectiveness in various pathological conditions [11]. Brazilian red propolis exhibits potent anti-inflammatory activity. RAW264.7 macrophages are induced by lipopolysaccharide (LPS) mimic nitric oxide synthesis, leading to a proinflammatory response [12]. Propolis blocks this subsequent action along with inhibiting Th-1- and Th-2-type T cells. These cells do not express their activity because of the strong affinity of propolis to suppress the generation of various types of cytokines and DNA synthesis along with the stimulation of transforming growth factor-β1 [13]. Zhang et al. examined the blocking potency of apigenin and suggested a remarkable decrease in interleukins in human-derived macrophages [14]. An in vivo study also supported the data obtained after 14 days of administration of propolis in mice [15]. Another cause of inflammation is highly influenced by the release of prostaglandin and leukotrienes, which are the byproducts of cyclooxygenase (COX) expression and lipoxygenase (LOX). Anatolian propolis intervenes in the action of COX, which blocks the further production of inflammatory cells [16]. A unique compound found in propolis is caffeic acid phenethyl ester (CAPE), which successfully blocks cytokine production and release as well as the multiplication of T cells by blocking nuclear factor-KB (NF-κB), leading to a decrease in COX-2 expression and NO inhibition [17]. Along with NF-κB, the phenolic components of propolis alter the transcription of signal transducer and activator of transcription 1 (STAT-1), neutralizing free radicals to exhibit antioxidant properties [18,19].

Major concern diseases, such as CVD, diabetes and allergies, are managed primarily through antioxidant, anti-inflammatory and immune-modulatory properties, which are all properties of propolis [20]. Reactive oxygen species (ROSs), under physiological conditions, are important in activities including protein synthesis, cell proliferation, inflammation and many more. Excess oxidative stress can lead to DNA damage and mutation, which can lead to tumor formation over time [21]. Propolis scavenges hydrogen peroxide, superoxide, hydroxyl ions and many more, along with increasing the gap junction for communication between cells. This helps to decrease ROSs and, thus, reduce oxidative stress [22]. Because of its antioxidant and anti-inflammatory action, it has potent hepatoprotective properties. Induced mercury toxicity was well handled by consecutive administration of propolis (200 mg/kg) in mouse livers, leading to the conclusion that propolis nullifies the toxicity of mercury and, thus, can be proven to be a potent hepatoprotective agent [23].

Tumor expansion can be regulated by controlling cell proliferation, especially targeting tumor cells. Propolis works on various mechanisms to inhibit its action. Propolis downregulates the formation of growth factors, vascular endothelial growth factors and signaling pathways such as IL and NF-κB, and also reduces the transcription of the cell cycle along with inducing apoptosis [24]. For the proliferation of cells, balance among cyclic-dependent kinases (CDKs), CDK inhibitors and cyclins is essential and is altered by propolis components. Kabala-Dzik et al. compared the effectiveness of CAPE along with caffeic acid against a human breast cell line and concluded that higher cytolysis and cell death were observed in CAPE than in CA [25]. Flavonoids of propolis extracts from Asian traditional medicine decrease the cellular division of the estrogen receptor (ER), which is essential in cancers such as breast, ovaries, endothelium (ER-α) and bone, vessels of blood, bone and lungs (ER-β) [26]. It also inhibits telomerase reverse transcriptase, which leads to distortion of telomeres and eventually cell death [27].

**Figure 1:**
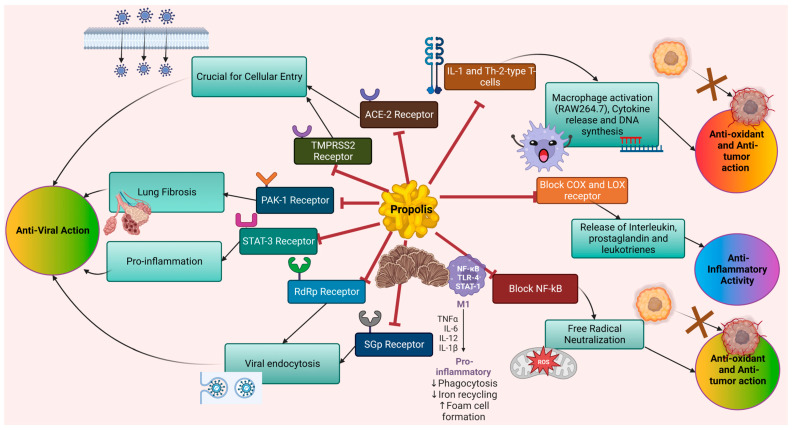
Propolis has potent antioxidant, antitumor, anti-inflammatory and antiviral activities. It inhibits various receptors that are responsible for the respective actions.

Propolis possesses antiviral properties against various DNA and RNA viruses, especially poliovirus (PV), herpes simplex virus [28], influenza virus [29], rhinovirus [30], SARS-CoV-2 [31,32] and many more (Figure 1). Propolis possesses potential inhibitory efficiency on protein kinase (PAK1), angiotensin-converting enzyme (ACE-2), STAT-3, RNA-dependent RNA polymerase (RdRp), transmembrane serine protease 2 (TMPRSS2) and spike glycoprotein (SGp), especially in the management of SARS-CoV-2, which shows potent antiviral properties [33,34,35]. ACE-2 and TMPRSS2 are responsible for cellular entry, PAK1 induces lung fibrosis, STAT-3 activates the proinflammatory response and RdRp and SGp mediate viral endocytosis [34,36]. It targets the entire viral cycle, ensuring effectiveness, and is widely used as a therapeutic molecule, along with being adjuvant to vaccination approaches.

Globally, diabetes is a major concern to the community, and various approaches are being developed to control the disease. Propolis works on various targets to treat diabetes mellitus (DM). Contributing modifications in the body, such as an exaggerated hexosamine pathway activated PF-C, increased glycation end products and oxidative stress, can elevate DM [37]. Propolis derived from the vicinity of Kayseri Province provides a strong antioxidant effect and reduces oxidative stress by inhibiting malonaldehyde levels, which leads to improved glucose metabolism [37,38,39]. Miranda et al. stated that Brazilian green propolis significantly reduced TNF-α, but did not interfere with other interleukins [40]. Algerian propolis is a miraculous bundle of bioactive constituent molecules that shows effectiveness against various diseases with few to no side effects. It has acceptable pharmacokinetic properties that facilitate its utilization [41].

## 3. Molecular Docking for Propolis Constituents against Chronic Diseases

Propolis has been found to have a wide range of pharmacological properties, including anti-infectious, antioxidant, anticancer, anti-inflammatory and immunomodulatory properties. The commonly studied phytoconstituents of propolis are quercetin, hesperidin, limonin, myricetin, kaempferol, CAPE, etc. We have also investigated the role of these constituents in various diseases using an in silico method. Molecular docking was carried out to understand the mechanism of propolis in COVID-19, tuberculosis and cancer. The study was performed using Schrödinger suite version 13.3 (Schrödinger LLC, New York, NY, USA, 2022). The 3D protein structures were retrieved from the RCSB Protein Data Bank [42] for established and validated targets such as main protease (Mpro) (PDB ID 7K40), spike glycoprotein—ACE2 receptor binding domain (spike ACE2 RBD) (PDB ID 6M0J), RNA-dependent RNA polymerase (RdRp) (PDB ID 7BV2) for COVID-19, enoyl acyl carrier protein reductase (InhA) (PDB ID 4TZK), decaprenyl-phosphoryl-ribose 2′-epimerase (DprE1) (PDB ID 4P8N) for tuberculosis, epidermal growth factor receptor (EGFR) (PDB ID 3POZ), phosphoinositide 3-kinase gamma (PI3K) (PDB ID 3L54) and caspase-3 (PDB ID 3DEI) for cancer.

All the crystal structures of protein were prepared using the Protein Preparation Workflow tool of Maestro, where they were minimized by optimization of hydrogen bonds. The phytoconstituents of propolis were sketched using ChemDraw and prepared using the LigPrep tool. The grid was generated using the Receptor Grid Generation module by tackling the centroid of co-crystallized ligands of all the protein structures. The GLIDE tool was used for extra precision (XP) docking. The docking protocol was validated by calculating all atom RMSD between the docked structure and co-crystallized ligand, which ranged between 0.37 and 0.09 Å for all protein structures.

**Figure 2:**
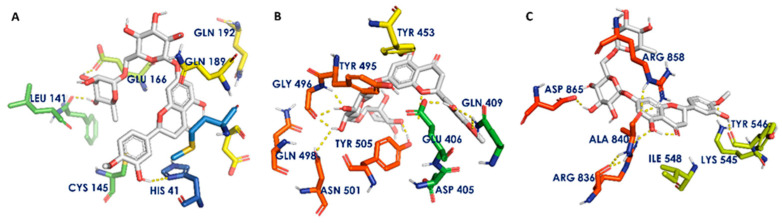
Receptor—ligand interaction diagrams. (**A**–**C**): Interaction of hesperidin with the active sites of Mpro, spike-ACE2 RBD and RdRp, respectively. Gray sticks indicate ligands, atom-type color sticks indicate amino acid residues of receptor and yellow dotted lines represent hydrogen bonds formed between ligands and receptors.

Hesperidin, myricetin, quercetin and kaempferol were found to have good docking scores against the selected protein structures of SARS-CoV-2. Table 1 describes the docking scores of the constituents. The receptor—ligand interaction diagrams of the best docked compound hesperidin against the promising targets of COVID-19 (docking score against Mpro: −9.59 kcal/mol, spike-ACE2 RBD: −9.25 kcal/mol and RdRp: −8.91 kcal/mol) are shown in Figure 2. Hesperidin interacts with Mpro by forming hydrogen bonds with His41 (key amino acid of the catalytic dyad), Phe140 and Glu166. It was also found to interact with the spike ACE2 RBD by forming hydrogen bonds with Glu406, Gln409, Gly496 and Gln498 amino acid residues. Hydrophilic interactions were also observed with RdRp by forming hydrogen bonds with Tyr546, Arg836, Ala840, Asp858 and Arg865. The other phytoconstituents were also observed to have similar interactions with SARS-CoV-2 proteins. Only CAPE showed very poor interaction with RdRp, possessing a docking score of −2.07 kcal/mol.

**Figure 3:**
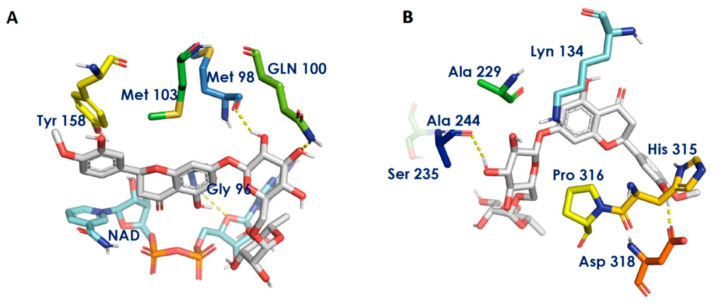
Receptor—ligand interaction diagrams. (**A**,**B**): Interaction of hesperidin with the active sites of InhA and DprE1, respectively. Gray sticks indicate ligands, atom-type color sticks indicate amino acid residues of receptor and yellow dotted lines represent hydrogen bonds formed between ligands and receptors.

*Mycobacterium tuberculosis* is the causative agent for tuberculosis. Mycolic acid is the active component of the bacterial cell wall and has become the most promising target to combat the disease. InhA and DprE1 are the key enzymes involved in the synthesis of mycolic acid [43]. Hesperidin, myricetin, quercetin and kaempferol showed good potential to inhibit these enzymes when investigated using molecular docking. Among all the constituents depicted in Table 2, hesperidin showed a good docking score against InhA (−11.95 kcal/mol) and DprE1 (−10.79 kcal/mol). It was found to interact with Met98 and Gln100 of InhA by forming hydrogen bonds (Figure 3A), as well as with Ala244 and Asp318 of DprE1 (Figure 3B). Limonin showed comparatively poor interactions with InhA and poorer interactions with DprE1. CAPE also poorly interacts with DprE1.

**Figure 4:**
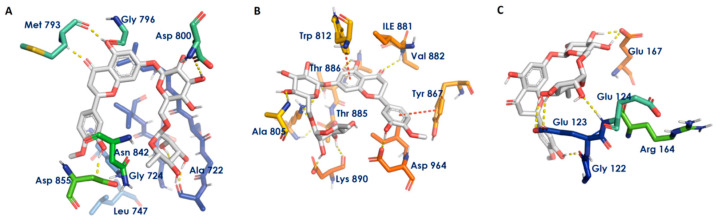
Receptor—ligand interaction diagrams. (**A**–**C**) Interaction of hesperidin with the active sites of EGFR, PI3K and caspase-3, respectively. Gray sticks indicate ligands, atom-type color sticks indicate amino acid residues of the receptor and yellow and red dotted lines represent hydrogen bonds. π–π stacking formed between the ligands and the receptor, respectively.

Propolis phytoconstituents have been associated with cell proliferation and tumor growth modulation pathways such as pI3k/Akt, EGFR and caspase [17]. In this case, one of the important constituents of propolis, hesperidin, was discovered to interact with the aforementioned receptors, as evidenced by its high binding affinity with these receptors. It exhibited docking scores of −13.43 kcal/mol against EGFR, −13.93 kcal/mol against pI3K and −7.84 kcal/mol against caspase-3 (Table 3). The hydroxyl and keto groups of hesperidin were found to interact with the Ala722, Gly724, Met793, Asp800 and Asp855 amino acid residues of EGFR by forming hydrogen bonds (Figure 4A). Hesperidin was observed to exhibit two π–π stacking interactions with the Trp812 and Tyr867 residues of pI3K. It was also found to form hydrogen bonds with Ala805, Val882, Lys890 and Asp964 of pI3K (Figure 4B). It was also observed that hesperidin forms hydrogen bonds with the Gly122, Glu123, Glu124 and Glu167 residues of caspase-3 (Figure 4C). Limonin was found to have less binding affinity for these three receptors than hesperidin.

As per the above molecular docking study, hesperidin was the best phytoconstituent of propolis involved in COVID-19, TB and cancer, and can be evaluated further for better therapeutic development.

## 4. Chronic Diseases with Reported Therapeutic Properties of Propolis and Its Active Constituents

Propolis has been effectively evaluated with medicinal importance in various chronic diseases. Brazilian propolis was found to reduce renal dysfunction by reducing proteinuria and oxidation stress levels [44,45]. Various varieties of propolis have been attributed to benefits in heart dysfunction, including the influence of inflammatory factors [46]. The anticancer activity of propolis and its constituents is commonly associated with blocking the localization of NF-κB, inducing ROS and regulating gene expression [47]. Propolis has potent antioxidant properties and beneficial effects on the inflammatory state as well as on oxidative stress conditions in RA patients [48]. The potential effects of propolis and its constituents against well-known chronic diseases are summarized below.

A summary of some well-known chronic diseases as well as propolis and its constituents against them.

### 4.1. Chronic Kidney Disease

CKD has been a well-known cause of suffering and mortality in recent decades [49]. CKD is observed with the majority of pathogenic complications involving inflammatory and oxidative stress conditions. Oxidative stress is said to be the main causative pathway for the progression of CKD [50]. While various medicaments are available for reducing and controlling complications arising in CKD, new remedies with enhanced medicinal benefits and few side effects are still in demand. In recent years, propolis has been investigated for its potential action regarding repairing kidney tissue injuries and returning normal kidney function in acute kidney injury and CKD [51]. Some of the important clinical trials examining the activity of propolis and its constituents against CKD have been summarized in Table 4. 

Silveira et al. studied a double-blinded, randomized, placebo-controlled model in CKD patients and concluded that Brazilian green propolis extract was capable of significantly reducing proteinuria in diabetic and non-diabetic CKD patients. A dose of 500 mg/day for 12 months of treatment resulted in a reduction in proteinuria and a significant decrease in urinary monocyte chemoattractant protein-1, but no change in estimated GFR or blood pressure [45]. Nada Oršolić et al. examined the effect of Croatian propolis extract in a diabetic mouse model and reported the healing action of propolis preparation on diabetic hepatorenal damage. This effect is proposed to occur through the antioxidant activity of propolis [52]. Ahad et al. examined the nephroprotective effects of chrysin (a bioactive constituent of propolis) in a STZ/HFD-induced T2DM Wistar albino rat model and revealed a reduction in oxidative stress and renal inflammation by chrysin by inhibiting the TNF-α pathway, restoring renal function [53]. Jessica Granados-Pineda et al. explored the preventive effect of pinocembrin (a bioactive constituent of Mexican brown propolis) using a diabetic nephropathy in vivo model. Pinocembrin shows a preventive effect on the progression of dyslipidemia and kidney damage. However, at advanced stages, acceleration of disease progression by pinocembrin was observed [54]. Wei Zhu et al. proposed the preventive property of propolis on hepatorenal injury through blocking peroxidation of lipids, and induction of antioxidant enzymatic activity upon observing the beneficiary effect of Brazilian propolis and Chinese propolis against diabetic Sprague Dawley rats induced with hepatorenal injuries [55]. Iranian propolis was shown to reduce blood glucose levels (BGLs), improve antioxidant levels and diminish histopathological changes in the T1DM rat model [56]. Teles et al. demonstrated renal protective effects in a severe CKD model with a reduction in kidney injury-related inflammation and oxidative stress conditions. Brazilian red propolis diminished the progression of proteinuria in 5/6 renal ablation model Wistar rats, partially prevented heart hypertrophy, and resulted in a 24% higher rate of survival compared to untreated rats [44]. Ulusoy et al. examined the healing property of propolis on methotrexate-induced renal damage by noting the amount of heat shock protein-70 expression returning to baseline levels with the improvement of nephrotoxicity [57]. The neuroprotective action of propolis in a rodent model was studied by Chang et al. who mentioned the inhibitory activity of propolis on SMAD 2/3-dependent pathways and further suppressed the activation of SMAD-independent JNK/ERK [58].

**Table 4:** Summary of clinical trials examining the activity of propolis and its constituents against CKD.

Propolis	Study Design	Dose	Measured Outcome	References
Brazilian green propolis extract	double-blinded randomized placebo-controlled study on CKD patients	500 mg per day for 12 months	Lowered the level of monocyte chemoattractant protein-1 in urine Arrested proteinuria significantly	[45]
Water-soluble derivative of Croatian propolis	Swiss Albino mice model	50 mg/kg per day for one week	Improved in body weight, immunological and hematological parameters Reduced degree of vacuolization, corpuscular, tubular changes Suppressed diabetic hepatorenal damage	[52]
Chrysin, propolis constituent	STZ/HFD-induced T2DM Wistar albino rat model	40 mg/kg per day for 16 weeks	Lowered the TNF-α expression Inhibited expression of fibronectin, TGF-β and NF-κB	[53]
Brazilian propolis and Chinese propolis	STZ-induced diabetic male Sprague Dawley rats	Group A = 100 mg/kg Chinese propolis and Group B = 100 mg/kg Brazilian propolis twice daily for 8 weeks	Enhancement in oxidative stress level Reversed level of serum alanine, transaminase, aspartate transaminase and urinary albumin excretion rate Increased in the SOD level by Chinese propolis	[55]
Ethanol extract of Iranian Propolis	STZ-induced diabetic Wistar rats	100 mg/kg per day and 200 mg/kg per day for 6 weeks	Decreased level of malondialdehyde Improvement in SOD and GPx activities	[56]
Brazilian Red Propolis	5/6 renal ablation model Wistar rats	150 mg/kg per day for three months	Reduced instances of glomerulosclerosis, Decreased level of serum creatinine retention, hypertension, proteinuria, oxidative stress and renal macrophage infiltration	[44]
Propolis, unknown source	Methotrexate-induced kidney injury in male Wistar albino rats	100 mg/kg per day for 15 days	Minimized the rise in the number of apoptotic cells; heat shock proteins-70 expression Improved kidney morphology	[57]
Taiwanese green propolis	Aristolochic acids-induced nephropathy model in C57BL/6 mice	0.2 mg/kg for 12 weeks	Improved tubulointerstitial fibrosis; facilitated renal excretion of p-cresyl sulfate and indoxyl sulfate	[58]

### 4.2. Rheumatoid Arthritis

Rheumatoid arthritis (RA) falls into the class of autoimmune diseases comprising oxidative stress and chronic inflammatory events. Inflammation progresses in arthritis due to NF-κB activation mediated through ROS [59,60]. Several factors have been reported to facilitate the release of IL-1, IL-6 and TNF-α-like proinflammatory cytokines [59]. Depressing inflammatory conditions may be beneficial in RA [61]. The suppressive effect of an ethanolic extract of propolis was estimated by Park et al. against an arthritis rat model, and they proposed an anti-inflammatory effect resulting from inhibition of prostaglandin production [62]. Orsi et al. demonstrated the non-specific immunological response of propolis associated with macrophage activation and nitric oxide inhibition [63]. Propolis can also prevent inflammation by hindering the NF-κB pathway and decreasing ROS levels [48]. The chemical composition of propolis has powerful anti-inflammatory properties capable of regulating immune cell functions and reducing the immune response mediated through cytokines [64]. CAPE is a flavonoid-like bioactive compound of propolis that specifically inhibits NF-κB activation [65]. Polyphenolic constituents found in propolis possess free radical scavenging activity against ROS [66], and the polyphenolic profile of propolis has a direct correlation with its antioxidant properties [67]. Tanaka et al. reported that the suppressive effects of Brazilian propolis possess beneficial effects in a mouse model of RA and proposed that the suppressive effect results from a reduction in IL-17 production by blocking the differentiation of IL-17-secreting cells, also denoted as Th17 cells [68]. However, a double-blinded, randomized, controlled trial performed by Matsumoto et al. mentioned the lack of beneficial activity of propolis in patients with RA [69].

### 4.3. Cancer

Various preclinical studies have reported the chemopreventive and anticancer properties of different types of propolis and its major constituents, such as flavonoids and polyphenols [70]. Table 5 summarizes such important clinical trials. Portuguese propolis has effective antiproliferative activity in human renal cell carcinoma (RCC), and free radical-induced erythrocyte damage is also protected by propolis [71]. CAPE (a bioactive constituent of propolis) was reported to have anticancer properties by inhibiting NF-κB, caspase and Fas signaling activation in MCF-7 cells [72]. Kamiya et al. revealed potential inhibition of MCF-7 breast cancer cells by Brazilian red propolis extract and CAPE. They concluded that the anticancer activity was due to the induction of DNA fragmentation, CCAAT/enhancer binding protein homologous protein expression and caspase-3 activity [73]. Xuan et al. suggested that the antitumor activity of the ethanol extract of Chinese propolis (EECP) and its bioactive constituents mainly persist due to regulation of the annexin A7 and p53 proteins, mitochondrial membrane potential and ROSs, as well as that inhibition of NF-κB causes apoptosis in cancer cells [74]. Chang et al. found that Chinese propolis and CAPE exert inhibitory effects on the proliferation and migration of the TLR4-positive MDA-MB-231 cell line. Action may be increased by TLR4 signaling pathway blockades, apoptosis and autophagy. Cell proliferation was significantly reduced by 25 μg/mL and 50–100 μg/mL CAPE and EECP [75]. The cytotoxic and antiproliferative potency of Cuban red propolis was determined by Herrera et al. and reported on the MDA-MB 231 tumor cell line, and the inhibitory effect of propolis was proposed to occur through the induction of mitochondrial dysfunction, resulting in ROS-associated necrosis [76]. A concentration of 100 μg/mL was able to attain 71% cytotoxicity [76]. The anticancer potency of Chinese poplar propolis was examined by Li et al. in a MDA-MB-231 cancer cell line stimulated with LPS in an inflammatory microenvironment [77]. They illustrated a propolis-mediated reduction in proinflammatory cytokine production and a negative effect on angiogenesis, proliferation and migration of tumor cells. A concentration of 25–200 μg/mL noticeably inhibited the metastasis of breast cancer [77]. Propolis collected from Poland was evaluated by Milena et al., and promising antiproliferative activity was found against colon, lung, breast and glioma cancer cell lines [78].

### 4.4. Diabetes

Diabetes mellitus (DM) is a chronic disease that arises from either a decline in insulin synthesis (type 1 diabetes mellitus, T1DM) or a decrease in insulin sensitivity (type 2 diabetes mellitus, T2DM) that leads to hyperglycemia. Recent studies (Table 6) have proven that elevated glycemic levels and other diabetic complications are well controlled by natural bioactive compounds and dietary supplements [79]. Yajing Li et al. observed improved insulin sensitivity and lipid levels in a T2DM rat model after treatment with encapsulated propolis, which was obtained from the *A. mellifera* L. species of honeybees found in northern China. A total of 100 male Sprague—Dawley rats were given a high-fat diet and streptozotocin to induce diabetes. Low (50 mg/kg), medium (100 mg/kg) and high (200 mg/kg) doses of encapsulated propolis were administered to T2DM rats, and 10 mg/kg pioglitazone was used as a positive control. The fasting blood glucose, fasting serum insulin and triglyceride levels were found to be decreased, which was comparable with the control, pioglitazone, although there was no change in total cholesterol or body weight [80]. Chihuahua propolis was evaluated for its hypoglycemic effect on a T2DM mouse model, and it was revealed to have antioxidant activities [81]. The beneficial effect of Brazilian propolis was examined by Aoi et al. on Otsuka Long-Evans Tokushima Fatty (OLETF) rats. Eight weeks of treatment resulted in the control of BGL and a decrease in insulin levels in blood. Furthermore, propolis also improved cardiovascular and metabolic complications [82]. Chen et al. showed that the ethanolic extract of Taiwanese green propolis plays a preventive role on damaged pancreatic β-cells in STZ- and HFD-induced T2DM rat models, as well as that disease progression can be retarded upon propolis treatment [83]. Brazilian propolis was found to protect mesenteric adipose tissues by regulating immune cell density and reducing the amplitude of diabetes [84].

Investigations of the potency of propolis and its beneficial effects on diabetes complications were also performed in a human diabetic model. Iranian propolis treatment for 8 weeks at 3 times the dose of 500 mg can control glucose levels and inflammatory conditions in T2DM patients [85]. A similar clinical study showed a significant decrease in fructosamine and oxidized LDL as well as an increase in catalase activity [86]. Fukuda et al. revealed an improvement in glomerular filtrating dysfunction by Brazilian green propolis, probably by resisting the worsening of uric acid levels, inflammation and GFR, which generally occur in T2DM patients [87]. Zhao et al. applied Brazilian green propolis treatment to T2DM patients and interpreted the elevation of patients’ antioxidant statuses [88]. Chinese propolis was reported to enhance the serum antioxidant framework and thereby improve antioxidant defensive activity in T2DM patients [89]. Samadi et al. examined the effects of 12 weeks of 300 mg doses of propolis administered three times daily, which could control lipid content and FBG by improving diabetic symptoms in patients [90]. Zakerkish et al. found improved glucose metabolism in T2DM patients; furthermore, their study also supported beneficial effects on renal and hepatic impairments [91].

**Table 6:** Summary of clinical trials examining the activity of propolis and its constituents against DM.

Propolis Source	Study Design	Dose	Measured Outcome	References
Encapsulated propolis, China	T2DM rat model	Low dose =50 mg/kg per day, middle dose =100 mg/kg per day and high dose= 200 mg/kg per day	No improvement in body weight and cholesterol level Decreased FBG, fasting serum insulin and triglyceride levels Improvements in insulin act index and insulin sensitivity	[80]
Mexican chihuahua propolis	STZ-diabetic mice model	0.3 g/kg per day, fifteen days of treatment	Improvements in body weight and plasma insulin levels Controlled the elevated BG Elevated the activities of enzymes that participate in the antioxidant system	[81]
Brazilian propolis	OLETF rats model	One group with 0.1% *w*/*w* and another group with 0.5% *w*/*w* propolis diet	No improvement in body weight Reduced plasma insulin levels and systolic blood pressure and a rise in pH of the interstitial compartment Reduced the elevated BGL only in the group treated with a 0.5% dose	[82]
Taiwanese green propolis ethanolic extract	STZ/HFD-induced T2DM rat model, three different groups: controlled group, 1X group and 5X group	183.9 mg/kg per day, 919.5 mg/kg per day for 8 weeks.	Maintained the density of β-cells in the pancreas and levels of inflammatory cytokines Induced genes related to lipid metabolism by the high propolis diet group	[83]
Brazilian propolis ethanolic extract	C57BL/6 male obese mice	100 mg/kg, two doses within seven days, twelve weeks of treatment	Light improvement in body weight and TC, enhanced insulin sensitivity and BGL Reduction in BGL and plasma cholesterol levels and macrophage numbers, and elevation of eosinophils in mesenteric tissues	[84]
Iranian propolis	Human clinical trial: randomized, double-blinded, controlled study on T2DM patients	500 mg propolis capsule three times per day for 8 weeks	Lowered levels of TNF-α, HbA1c and 2-hp Reduced levels of FBG and C-reactive protein No improvement in hepatic enzyme activity	[85]
Brazilian green propolis	Human clinical trial: randomized, double-blinded, controlled study on T2DM patients	226.8 mg every day for 8 weeks	No improvement in insulin and glucose levels Maintained GFR, TNF-α and uric acid at normal levels	[87]
Brazilian green propolis	Human clinical trial	900 mg every day for 18 weeks	Decreased levels of inflammatory mediators, serum carbonyls and LDH	[88]
Chinese propolis	Human clinical trial, randomized controlled study	900 mg every day for 18 weeks	No improvement in glucose metabolism, TNF-α or serum carbonyls levels Elevated levels of glutathione and constituents of propolis excreting antioxidant properties Decrease in lactate dehydrogenase activity and amount of LDH in serum	[89]
Bee propolis peels manufactured by Soren Tech Toos, Mashhad, Iran	Human clinical trial: randomized, double-blinded, controlled study on T2DM patients	300 mg propolis pills three times per day for 12 weeks	Decreased FBG with better control of TC and LDL levels	[90]
Iranian propolis	Human clinical trial: randomized, double-blinded, controlled study on T2DM patients	500 mg Iranian propolis capsule twice a day for 12 weeks	Diminished in FBG, HbA1c, insulin resistance, 2 h post prandial, blood urea nitrogen and liver transaminase and inflammatory mediators Elevated level of HDL and maintain GFR	[91]

### 4.5. Tuberculosis

Tuberculosis (TB) is a chronic contagious respiratory infection in which the causative pathogen *Mycobacterium tuberculosis* (Mtb) infects the lung. The treatment of drug-resistant TB is often incurable, and new strategies are now a worldwide concern. Constituents of propolis showing antitubercular activity are promising new therapies to treat tuberculosis [92,93]. Scheller et al. correlated the infectious effects of various mycobacterial strains and the antitubercular potency of the ethanolic extract of propolis (EEP), where seven strains were found to be susceptible to propolis treatment [94]. EEP was established to have a synergistic effect upon enhancing the effectiveness of anti-tuberculosis drugs [95]. Essential oil obtained from green propolis in Brazil is composed of important chemical constituents [96]. This essential oil was evaluated by Quintino et al. and showed inhibitory potency against Mtb, with MIC= 64 μg/mL and MIC = 62.5 μg/mL found for *Mycobacterium avium* [97]. Volatile oil from brown propolis possesses promising antimycobacterial activity against Mtb and *M. avium,* with MIC = 50 μg/mL and 62.5 μg/mL, respectively [98]. Sawicki et al. observed metabolic and transcriptomic changes in Mtb by conducting an in vitro study of an ethanolic extract of Nepalese propolis, with MIC = 8 μg/mL for the Trigona species and MIC = 32 μg/mL for the mellifera species [99].

### 4.6. COPD

Chronic obstructive pulmonary disease emerges with inflammatory symptoms associated with the respiratory pathway. These are characterized by resistance to airflow due to abnormalities in the respiratory path upon exposure to toxic gases or particles [100]. Machado et al. revealed a protective effect of Brazilian green propolis on respiratory inflammation in a mouse model [101]. Investigations of herbal products in treating COPD have increased [102]. Khayyal et al. examined two months of treatment with silver sachets (propolis extract) in asthma patients and showed improvement in immunological parameters, severe attacks and pulmonary dysfunction in patients [103]. Kolarov et al. studied the beneficial effect of propolis in combination with N-acetylcysteine, which was able to suppress cough in COPD patients [104]. This combination was proposed to suppress the development of coughing and, thereby, reduce the incidence of severe attacks in COPD patients [105].

### 4.7. Cardiovascular Disease

CVD is related to dysfunction of the heart and blood circulation, and is a rising threat with major morbidity as well as health and economic burdens globally. It is elevated in conditions such as angina, stroke, rhythm and heart failure [106]. Reduction in diameter and resisting flow of blood in blood vessels are general conditions of CVD [107]. Table 7 illustrates the beneficial effects of propolis and its constituents in CVD. Balion et al. explored the inhibitory property of propolis on mitochondrial respiration in a rat model. Aqueous (AqEP), polyethylene glycol-aqueous (Pg-AqEP), and ethanolic propolis extracts (EEP) have been shown to influence mitochondrial and ROS production in astrocytic C6 cells. PC EEPs (2 to 4 g/mL) provided less protracted protection against ischemia-induced superoxide generation and mortality. Pg-AqEP and Ag-EP (but not EEP) both effectively protected cultures from hypoxia-induced increases in TNF, IL-1, and IL-6. Only Pg-AqEP (not AqEP or EEP) reversed the hypoxia-induced reduction in mitochondrial basal and ATP-coupled respiration rates while also dramatically increasing mitochondrial respiratory capacity. The extent of the inhibitory effect is dose-dependent and affected by substrates utilized for respiration [108]. Zhang et al. examined the in vitro anti-platelet aggregation action of a water extract of propolis in a dose-dependent manner [109]. Majiene et al. explored the inhibitory property of propolis on mitochondrial respiration in a rat model. The extent of the inhibitory effect is dose-dependent and affected by substrates utilized for respiration [108]. Wang et al. proposed a beneficial effect of the total flavonoid constituents of propolis by regulating the expression of connexin 43 on CHF in a rat model [110]. Chao et al. examined propolis treatment against carotid restenosis with improvement in disturbed cardiac functions [111]. They looked at how propolis (125 and 250 mg kg^−1^ day^−1^) affected carotid restenosis in hypercholesterolemic rabbits. In hypercholesterolemic rabbits, propolis dramatically decreased the degree of carotid restenosis, prevented neointima hyperplasia, lowered blood lipid profiles, and boosted antioxidative activity. Furthermore, propolis decreased the expression of CD68, TLR4, NF-B p65, MMP-9 and TNF- in the carotid arteries, and also lowered plasma levels of C-reactive protein, interleukin-6 and TNF-. Propolis has a protective impact on carotid restenosis in rabbits, which is connected with controlling blood lipids and decreasing oxidative stress and inflammation, and its anti-inflammatory action may be related to suppressing the TLR4-mediated NF-B signaling pathway. In ApoE-/- mice, a 14-week combined therapy of simvastatin (a cholesterol-lowering medicine) and pinocembrin dramatically lowered blood lipid levels, enhanced endothelial function and reduced atherosclerotic symptoms [112]. Platelet aggregation has been linked to atherosclerosis and atherothrombosis, two complicated multifactorial chronic inflammatory illnesses caused by fat and other macromolecule buildup in artery walls. CAPE (15 and 25 M) substantially reduced collagen-stimulated platelet aggregation. It was reported that CAPE exhibited cardioprotective effects in short-term myocardial ischemia in rats through a reduction in xanthine oxidase (XO) and adenosine deaminase (ADA) activities, as well as antioxidant effects [106,113].

### 4.8. Other Chronic Diseases

Propolis has many active constituents and is utilized for medicinal value in many diseases [115]. Chen et al. reported that a small molecule extracted from green propolis improved metabolic syndrome in a glycemic mouse model by influencing hepatic glucose metabolism and lowering the levels of glucose and lipids [116]. The antiviral potency of propolis against SARS-CoV-2 in vitro was determined by Refaat et al., who reported a potency comparable to that of remdesivir [117]. A molecular docking study showed that constituents of ethanolic propolis extracts are capable of having of inhibitory activity against ACE-2 receptors for SARS-CoV-2, especially rutin, which exhibits the best inhibition potential of −8 kcal/mol binding energy [118]. Khayrani et al. performed a similar molecular study with Sulawesi propolis compounds, where five constituents were found to have high potential affinity for ACE-2 receptors [119]. Egyptian propolis has also been reported as a promising candidate in combatting against SARS-CoV-2 using computational modeling [120]. Several other computational studies reported those supporting the affinity of propolis constituents toward SARS-CoV-2 [121,122,123]. The bioactive constituent of propolis CAPE has been proven to have a neuroprotective effect in in vivo models of Parkinson’s disease [124]. Zulhendri et al. summarized the therapeutic value of propolis on the brain regarding neurological disorders and injuries, suggesting that its suppressive effect on oxidative stress and inflammatory mediators also influences protein-coding gene expression and reduces apoptosis in various disorders [125].

## 5. Current Scenario of Preclinical and Clinical Development

Propolis is highly accepted for the treatment of various chronic diseases (Table 8). A preclinical study conducted in 36 mice regarding the management of high diet-based hypertension concluded that when propolis was administered for a week, it showed significant therapeutic efficacy. Propolis promotes wound healing in just 3 days and increases collagen and fibroblasts at higher concentrations when compared to the control group [109]. Moura et al. concluded that propolis attains characteristics such as angiogenesis and fibroproliferative processes by studying a sponge implanted animal model [126]. Mesenteric adipose tissue plays a crucial role in the evolution of T2DM and is significantly targeted by propolis and its derivative. Ichi et al. suggested a noticeable reduction in weight by decreasing the adipose tissue reservoir in obese mice [127]. The remarkable anti-inflammatory activity of artepellin c, found in Brazilian green propolis, was cited by Paulino et al. as one of the main causes of damage to the lungs and other organs. They outlined their preclinical findings on rat paw edema. The average reduction in inflammation stimulated by carrageenan was reduced by 38% within 6 h [128]. With the support of much preclinical data, clinical trials have become crucial to ensuring therapeutic effects similar to those observed in other animals. The following demonstrates the clinical trials intended to prove the efficacy of propolis in numerous diseases.

## 6. Conclusions and Future Directions

Propolis is a wondrous molecule with a broad spectrum of action. It consists of over 300 active phytochemicals with a wide variety of effects on various chronic diseases. CAPE, rutin, quercetin, CA and many important chemical moieties are present in propolis, which helps in the management of various diseases, such as DM, CVD, allergy to SARS-CoV-2 and inflammation. Additionally, it is also beneficial for gastrointestinal disorders (giardiasis anti-H. pylori), gynecological care (recurrent vulvovaginal candidiasis) and oral health (halitosis gingivitis). Numerous clinical trials are ongoing on propolis. As a known molecule, it has a wide spectrum of exploration in the treatment of many diseases. It has certain limitations, such as allergic reaction (respiratory or epidermal symptoms), mouth ulceration and irritation, but they are not very common. In the future, a more detailed study on propolis and its incorporation in the treatment of various diseases will be required, from the smallest inflammation to life-threatening diseases such as cancer. It is crucial to attain critical knowledge related to its active constituents, its interaction with numerous receptors and its subsequent pharmacological actions. The long-term effect of propolis administration is of keen interest, and must be determined before developing it into a potential therapeutic moiety.

## Figures and Tables

**Table 1:** In silico anti-SARS-CoV-2 potential of propolis constituents.

Sr. No.	Name of Constituents	Docking Score (kcal/mol)		
		Mpro (PDB: 7K40)	Spike-ACE2 RBD (PDB: 6M0J)	RdRp (PDB: 7BV2)
1	Hesperidin	−9.59	−9.25	−8.91
2	CAPE	−8.84	−4.33	−2.07
3	Myricetin	−8.24	−9.51	−8.59
4	Quercetin	−8.20	−8.90	−8.65
5	Kaempferol	−7.90	−6.57	−7.98
6	Limonin	−7.12	−8.78	−6.42

**Table 2:** In silico anti-tuberculosis potential of propolis constituents.

Sr. No.	Name of Constituents	Docking Score (kcal/mol)	
		InhA (PDB: 4TZK)	DprE1 (PDB: 4P8N)
1	Hesperidin	−11.95	−10.79
2	CAPE	−10.12	−6.02
3	Myricetin	−9.64	−9.56
4	Quercetin	−10.89	−8.95
5	Kaempferol	−8.11	−8.53
6	Limonin	−6.82	−2.99

**Table 3:** In silico anticancer potential of propolis constituents.

Sr. No.	Name of Constituents	Docking Score (kcal/mol)		
		EGFR (PDB: 3POZ)	PI3K (PDB: 3L54)	Caspase-3 (PDB: 3DEI)
1	Hesperidin	−13.43	−13.93	−7.84
2	CAPE	−10.13	−6.76	−5.51
3	Myricetin	−10.59	−12.35	−5.20
4	Quercetin	−10.12	−10.49	−4.07
5	Kaempferol	−9.37	−10.04	−3.88
6	Limonin	−3.44	−2.96	−3.13

**Table 5:** Summary of clinical trials examining the activity of propolis and its constituents against cancer.

Propolis Source	Study Design	Dose	Measured Outcome	References
Portuguese propolis, Bornes and Fundão regions	RCC model	Hemolytic protection IC_50_, Bornes = 6.3 ± 0.7 μg/mL Fundão = 10.4 ± 2.7 μg/mL Anticancer activity IC_50_ Bornes = 56.5 ± 16.7 μg/mL Fundão = 56.1 ± 20.9 μg/mL	Reduced malondialdehyde by blocking lipid peroxidation and controlled hemolytic activity due to peroxy free radicals Dramatically controlled the growth of cancer cells	[71]
CAPE	MCF-7 cell line	IC_50_ = 10 μg/mL	Decreased the expression of X-chromosome-linked inhibitor of apoptosis, dose-dependent inhibition of tumor density Increased level of Bax protein cells Facilitated JNK activation in a time-dependent manner	[72]
Brazilian red propolis, CAPE	MCF-7 tumor cells and human fibroblasts	Propolis = 0.1–20 μg/mL CAPE = 0.1–2 μM	Induced in caspase 3 signaling pathway and improved DNA fragmentation, changes in expression of apoptotic proteins and enhancer-binding protein Negligible effect in fibroblasts	[73]
Chinese propolis ethanolic extract	MDA- MB-231, MCF -7 tumor cell line	25, 50, 100, 200 μg/mL	Lowered NF-κB and p65 levels Significantly enhanced levels of p53 and annexin A7 Induced ROS and apoptosis; decreased cancer proliferation	[74]
Chinese propolis ethanolic extract, CAPE	MDA- MB-231 tumor cell line	Propolis extract = 25 μg/mL (low dose), 50 μg/mL (middle dose) and 100 μg/mL (high dose) CAPE = 25 μg/mL	Arrested the production of nitric oxide and the growth of tumor cells Activated PARP enzyme and caspase-3 signaling pathways. Lowered the levels of NF-κB, p65 and TLR4 signaling pathways	[75]
Cuban red propolis	MDA- MB-231 tumor cell line	IC_50_ = 67.3 μg/mL	Disturbed the mitochondrial potential, lactate dehydrogenase released, production of ROS and cell migration Decline in expression of genes inducing apoptosis Inhibited functions of KRK1/2 and PI3 K/Akt pathways	[76]
Chinese poplar propolis	MDA- MB-231 tumor cell line	Low dose = 25 μg/mL, middle = 50 μg/mL and high = 100 μg/mL	Decreased cell tube generation, IL-6, IL-1β, TNF-α-like inflammatory mediators, glycolytic enzymes and mitochondrial potential Promoted ROS generation	[77]

**Table 7:** Summary of clinical trials which experimented with the activity of propolis and its constituents against CVD.

Propolis Source	Study Design	Dose	Measured Outcome	References
Water extract of propolis (CAPE and other flavonoids)	In vitro, platelet-rich plasma	25 to 300 mg/L	Blocked the process of platelet aggregation	[109]
Propolis water solution (propolis from Lithuania)	Male Wistar rat model	63 and 125 μg/mL	Reduced rate of cardiac mitochondrial state 2 respiration with NAD-specific pyruvate and malate substrate Reduced rate of cardiac mitochondrial state 3 respiration with FAD-specific succinate substrate No change in outer mitochondrial membrane	[108]
Total flavonoids of propolis	In vivo, chronic heart failure (CHF) model rats	Low, middle and high doses continuing for 6 weeks of treatment	Improved expression of protein connexin 43 Declined level of inflammatory cytokines; apoptosis in heart cells	[110]
Total flavonoids of propolis	In vivo, mice model	25 mg/kg per day and 50 mg/kg per day continuing for 7 days of treatment	Reduced body weight with maintained lipids content	[114]
Populus (raw material of propolis, north China)	Hypercholesterolemia rabbit model	125 mg/kg per day, 250 mg/kg per day, continuing for 6 weeks of treatment	Reduced body weight with maintained lipids content, declined level of inflammatory cytokines	[111]

**Table 8:** Various clinical trials have been conducted to ensure the efficacy of propolis against various diseases.

Other IDs	Conditions	Phase	Study Design	Number Enrolled	NCT Number
FJ2011-1.1	Stable angina pectoris	Phase 3	Randomized, parallel assignment, double	478	NCT01453582
31099320.6.0000.0049	COVID-19 Inflammation	Phase 2 Phase 3	Randomized, parallel assignment, quadruple	200	NCT04800224
2015-c03	Diabetes mellitus, Type 2	Not available	Case—control, cross-sectional	31	NCT03649243
Denise Mafra8	Chronic kidney diseases Inflammation	Not applicable	Randomized, crossover Assignment, triple	60	NCT04411758
E.6289	Oral mucositis	Not applicable	Randomized, parallel assignment, open label	64	NCT05250661
UFPa-0011	Dentin sensitivity	Not applicable	Randomized, parallel assignment, triple	18	NCT05083052
19-10-1269’	Endometriosis; peritoneum Endometriosis	Early phase 1	Randomized, parallel assignment, open label	120	NCT04374006
TabletUFRJPed	Dental plaque	Phase 2 Phase 3	Randomized, crossover assignment, triple	30	NCT03394729
00001	SARS-CoV-2 infection	Not applicable	Randomized, parallel assignment, open label	45	NCT04916821
UPlymouth	Blood pressure Periodontal diseases	Not applicable	Non-randomized, parallel assignment, quadruple	45	NCT04117451
DeniseMafra13	Chronic kidney diseases Inflammation	Not applicable	Randomized, parallel assignment, triple	34	NCT05183737
Propolis in 2 vehicles	Early childhood caries	Phase 2	Randomized, parallel assignment, single	60	NCT03812315
Hospital São Rafael S.A	Chronic kidney disease requiring chronic dialysis	Phase 1 Phase 2	Randomized, sequential assignment, open label	40	NCT04072341
AAkber	Dentine hypersensitivity	Early Phase 1	Randomized, parallel assignment, investigator	52	NCT04754763
54326916.4.0000.0068 USP Brazil	Chronic kidney diseases	Phase 2	Randomized, parallel assignment, triple	32	NCT02766036
IRB/ KKUCOD/ ETH/2018-19/112	Dental root sensitivity	Not applicable	Randomized, parallel assignment, double	75	NCT05588518
MDSJUZER ISRCTN66816132	Pain, post-operative	Phase 3	Randomized, parallel assignment, double	80	NCT03723980
MShah	Dentin hypersensitivity	Phase 2 Phase 3	Randomized, parallel assignment, single	40	NCT04819867
VSantos-UFMG	Dental plaque Gingivitis	Phase 2	Single group Assignment, open label	25	NCT01142843
339888	Radiation-induced mucositis of oral mucous membranes	Phase 2	Randomized, parallel assignment, triple	20	NCT01375088
10726612.8.0000.5149	Streptococcal infections Saliva altered	Phase 1 Phase 2	Single group assignment, open label	11	NCT02052973
DENT-1971	Diabetes mellitus Periodontitis	Phase 4	Randomized, parallel assignment, double	52	NCT02794506
FSoomro	Post-operative pain	Not applicable	Randomized, parallel assignment, double	90	NCT05569590
Propolis-metformin-T2DM	Diabetes mellitus, type 2	Phase 2	Randomized, parallel assignment, double	36	NCT0341612
31099320.6.0000.0048	Covid-19	Phase 2 Phase 3	Randomized, parallel assignment, single	120	NCT04480593
SSirry	Post-operative pain, chronic Necrotic Pulp	Not applicable	Randomized, parallel assignment, single	46	NCT04983524
1057529	Candidiasis, oral stomatitis, denture	Phase 3	Randomized, parallel assignment, open label	40	NCT02818803
oral Aphthosis 650/3607	Oral Mucositis ORAS Oral Infection Oral Ulcer	Early phase 1	Randomized, parallel assignment, triple	100	NCT05413096
oper252215	Caries, dental risk reduction	Not applicable	Randomized, parallel assignment, quadruple	64	NCT03553628
2022/61	Oral mucositis	Not applicable	Randomized, parallel assignment, triple	108	NCT05400031

## Data Availability

None added.

## Abbreviations

AAPH	2,2′-azobis(2-amidinopropane) dihydrochloride
ACE-2	Angiotensin-converting enzyme-2
BGL	Blood glucose level
CAPE	Caffeic Acid Phenethyl Ester
COPD	Chronic obstructive pulmonary disease
CKD	Chronic kidney disease
CVD	Cardiovascular disease
COVID-19	Coronavirus disease 2019
DM	Diabetes mellitus
DNA	Deoxyribonucleic acid
EEP	Ethanolic extract of propolis
FBG	Fasting blood glucose
GFR	Glomerular filtration rate
GPx	Glutathione peroxidase
HDL	High-density lipoprotein
HFD	High-fat diet
HOMA-IR	Homeostasis model assessment-insulin resistance
IL-1	Interleukin-1
IL-6	Interleukin-6
IL-17	Interleukin-17
IC_50_	Half-maximal inhibitory concentration
JNK	C-Jun N-terminal kinases
LDL	Low-density lipoprotein
LPS	Lipopolysaccharide
MCF	Michigan Cancer Foundation
Mpro	Main protease
Mtb	*Mycobacterium tuberculosis*
NF-κB	Nuclear factor kappa Β
OLETF	Otsuka Long-Evans Tokushima fatty
PE	Propolis extract
PARP	Poly-ADP ribose polymerase
RA	Rheumatoid arthritis
RCC	Renal cell carcinoma
ROS	Reactive oxygen species
SARS	Severe acute respiratory syndrome
SOD	Serum superoxide dismutase
STZ	Streptozotocin
T1DM	Type 1 diabetes mellitus
T2DM	Type 2 diabetes mellitus
TB	Tuberculosis
TC	Total cholesterol
TGF-β	Transforming growth factor-β
TLR4	Toll-like receptor 4
TNF-α	Tumor necrosis factor alpha
